# CircFBXW7 Inhibits Proliferation, Migration, and Invasion of Nonsmall Cell Lung Cancer Cells by Regulating miR-492

**DOI:** 10.1155/2022/8699359

**Published:** 2022-09-08

**Authors:** Shen-Yu Zhu, Zu-Xiong Zhang, Liang Gu, Ding-Yu Rao, Xiao-Yuan Tang, Cheng-Peng Sang, Yao Xia, Mao-Yan Si, Hua-Qiu Shi, Jie Li, Shu-Lin Li, Chun-Fa Xie, Xiao-Liang Yuan, Zhi-Xian Tang

**Affiliations:** ^1^Department of Thoracic Surgery, The First Affiliated Hospital of Gannan Medical University, Ganzhou 341000, China; ^2^Department of Pulmonary and Critical Care Medicine, The First Affiliated Hospital of Gannan Medical University, Ganzhou 341000, China; ^3^Gannan Medical University, Ganzhou 341000, China; ^4^Department of Oncology, The First Affiliated Hospital of Gannan Medical University, Ganzhou 341000, China

## Abstract

**Background:**

CircFBXW7 has been determined to be involved in various cancers; however, its role in nonsmall cell lung cancer (NSCLC) remains unclear. This study examined the function and potential mechanism of circFBXW7 in NSCLC.

**Methods:**

The structure of circFBXW7 was verified via RT-PCR and Sanger sequencing. The expression of circFBXW7 in NSCLC was determined by qRT-PCR. The effect of circFBXW7 overexpression on the proliferation, migration, and invasion of NSCLC cells was examined by CCK-8 and Transwell assays. Furthermore, a circFBXW7-miRNA network was established to explore their interaction. Predicted miRNA was determined by qRT-PCR. Moreover, the miRNA mimics were synthesized, wherein its effect on proliferation, migration, and invasion of NSCLC cells overexpressed circFBXW7 was assessed.

**Results:**

The circularity of circFBXW7 was verified. The expression of circFBXW7 was found to be downregulated in NSCLC cells compared with that in normal human lung epithelial BEAS-2B cells. Overexpression of circFBXW7 reduced cell proliferation, migration, and invasion. Furthermore, according to the circFBXW7-miRNA network prediction and qRT-PCR validation, miR-492 was identified to be the target of circFBXW7. The inhibitory effect of circFBXW7 overexpression on cell proliferation, migration, and invasion was reversed by miR-492 mimics.

**Conclusion:**

CircFBXW7 is downregulated in NSCLC. CircFBXW7 inhibits NSCLC cells proliferation, migration, and invasion by regulating miR-492.

## 1. Introduction

Lung cancer is the leading cause of cancer-related death, and nonsmall cell lung cancer (NSCLC) accounts for 85% of lung cancer mortality [1]. Although the survival of lung cancer has increased due to early diagnosis and improved treatment, its mortality remains high [2]. To improve the treatment of NSCLC, a better understanding of the molecules involved in NSCLC and their potential mechanism of action is needed to find new reliable biomarkers and therapeutic targets.

Circular RNAs (circRNAs) are a class of regulatory noncoding RNA with circular covalently bonded structures [3]. CircRNAs differ from linear RNAs as they are more stable with high tolerance to exonuclease digest, particularly in tissues and cells [1, 3]. Therefore, it is valuable to examine whether circRNAs are reliable molecules for cancer diagnosis and treatment [4]. Many circRNAs are conserved among eukaryotes. They localize in specific subcellular compartments and play different biological roles. Accumulating evidence shows that circRNAs regulate a diversity of cellular processes by acting as miRNA sponges, anchors for circRNA binding proteins (cRBPs), transcriptional regulators, molecular scaffolds, and sources for translation of small proteins/peptides. The understanding of the biological functions of circRNAs has brought a new perspective to our appreciation of cellular physiology and disease pathogenesis. Recent studies have shown that the expression of circRNAs is tissue and cell type-specific and is regulated in development or disease progression, where they exert specific biological functions [5]. Furthermore, circRNAs have been reported to regulate cancer progression by acting as competing endogenous RNA (ceRNA) and sponges for microRNA (miRNA) to control mRNA expression at the posttranscriptional level [1, 6]. For instance, circGDI2 was reported to regulate the miR-454-3p/FOXF2 axis in the process of proliferation, migration, invasion, and apoptosis in oral squamous cell carcinoma [7]. Several studies have shown that circ-SATB2 and circ-FGFR1 regulated cell proliferation, migration, and invasion via the miR-326/FSCN1 and miR-381-3p/CXCR4 axes, respectively [1, 8]. In addition, many studies confirmed that circRNAs are differentially expressed in patients with different stages of lung cancer and affect tumor recurrence and metastasis by targeting downstream miRNAs. Recently, researchers observed that circHMGB2 drives immunosuppression and antiPD-1 resistance in lung adenocarcinomas and squamous cell carcinomas via the miR-181a-5p/CARM1 axis [9].

CircFBXW7 is derived from the parent gene of F-box and WD repeat domain containing 7 (*FBXW7*) [10], which participates in the progression of cancer via the ceRNA mechanism and can be translated into protein. Yang et al. observed that circFBXW7 encodes an FBXW7-185 protein of 21 kDa that acts as a tumor suppressor in the human brain [11]. Gao et al. revealed that overexpression of circFBXW7 inhibits glioma proliferation and metastasis via the miR-23a-3p/PTEN axis [12]. However, the role of circFBXW7 in NSCLC has not been reported.

MiRNAs are short noncoding RNAs with about 22 nucleotides in length that are involved in the posttranscriptional regulation of gene expression [13]. MiRNAs play important roles in various biological processes, and abnormal miRNA expression is associated with diseases, including cancer [14]. Shi et al. reported that miR-19a promoted proliferation and migration of NSCLC cells by inhibiting PTEN expression [15]. Silencing miR-6515-3p has been shown to inhibit the proliferation of NSCLC cells [16]. Overexpression of miR-30c and miR-30d-3p inhibited the epithelial-to-mesenchymal transition (EMT) in NSCLC cells [17]. Besides, it has been reported that high expression of miR-492 was associated with poor prognosis of NSCLC patients. MiR-492 inhibitor inhibited the proliferation, migration, and invasion of tumor cells [18].

In this study, we aimed to examine the function and potential mechanism of action of circFBXW7 in NSCLC. Through bioinformatics analysis and validation, we identified miR-492 as the downstream target gene of circFBXW7 in NSCLC. The results of this study will provide potential use of circFBXW7 in the diagnosis and treatment of NSCLC, which will provide new ideas and targets for NSCLC research.

## 2. Methods

### 2.1. Cell Culture

Normal human lung epithelial BEAS-2B cells were obtained from Cellcook (Guangzhou, China) and were cultured in BEGM (#CM2010, Cellcook). NSCLC cells (HCC827, NCI-H358, NCI-H23, and NCI-H1299) were purchased from the Cell Bank of the Chinese Academy of Sciences (Shanghai, China). HCC827, NCI-H358, NCI-H23, and NCI-H1299 were cultured in RPMI-1640 (GIBCO, Grand Island, NY, USA), supplemented with 10% fetal bovine serum (FBS, GIBCO) at 37°C in a humidified atmosphere and 5% CO_2_. Besides, NSCLC PC9 cells were obtained from Cellcook (Guangzhou, China) and were cultured in RPMI-1640. All cells were passaged three times before the experiments.

### 2.2. Cell Transfection

The miR-492 mimics, circFBXW7 overexpression plasmids, and negative control were provided by Shanghai GenePharma Co., Ltd. (Shanghai, China). The mimics or plasmids were transfected into NCI-H23 and NCI-H1299 cells using Lipofectamine 2000 (Invitrogen, Thermo Fisher Scientific).

### 2.3. Reverse Transcription-Polymerase Chain Reaction (RT-PCR) and Real-Time Quantitative-PCR (qRT-PCR)

RNA was extracted from cells using TRIzol (Thermo Fisher) reagents and was used to produce cDNA via a cDNA Synthesis Kit (Takara, Japan). For reverse transcription (RT)-PCR, divergent primers and convergent primers were used to amplify products for agarose gel electrophoresis using 2× Taq Master Mix (Vazyme, Nanjing, China). The products of divergent primers of circFBXW7 were then Sanger sequenced. For qRT-PCR, AceQ® Universal SYBR qPCR Master Mix (Vazyme) was used to test the gene expression on an ABI 7500 real-time PCR instrument. The primers used in this study are shown in Table 1. *GAPDH* was used as the internal control for circRNA and mRNA expression, and *U6* was used as the internal control for miRNA expression. The relative expression of genes was calculated using the 2^−△△Ct^ method.

### 2.4. Cell Counting Kit-8 (CCK-8) Assay

After transfection for 48 h, cells were digested by trypsin (Gibco) and then were seeded into a 96-well plate at a density of 4 × 10^3^ cells per well. CCK-8 (Sigma) was added to each well after 24, 48, and 72 h. After 3 h, the light absorbance value at 450 nm was measured using a microplate reader (Beckman Coulter, USA).

### 2.5. Colony Formation Assay

After transfection for 48 h, cells were digested by trypsin (Gibco) and then seeded into a 6-well plate at a density of 600 per well. Cells were cultured at 37°C in a humidified atmosphere of 5% CO_2_ for 2 weeks. Then, cells were fixed for 30 min using formaldehyde and stained for 20 min using 0.1% crystal violet. After that, the plates were air-dried and the number of cell colonies was counted.

### 2.6. Transwell Assay

Cells were seeded into the upper chamber of a 24-Transwell system at a concentration of 4 × 10^4^ cells per well in a 200 *μ*L medium. Filters were precoated with Matrigel for testing cell invasion ability; 600 *μ*L RPMI-1640 medium plus 10% FBS was added into the bottom chamber. After 48 h, migrated cells were fixed for 30 min using formaldehyde and stained for 20 min using 0.1% crystal violet. An Olympus CKX53 inverted microscope (Olympus, Japan) was used to examine cell migration.

### 2.7. Network Construction

Potential miRNAs of circFBXW7 were predicted through miRanda software and miTarBase database, and Cytoscape software was performed to plot the circRNA-miRNA regulatory network. In addition, miR-492 was reported to regulate NSCLC progression [18–20].

### 2.8. Bioinformatics Prediction and Dual-Luciferase Assay

miRanda predicted the binding site of circFBXW7 and miR-492. CircFBXW7 sequences containing potential wild-type (WT) or mutant (MUT) binding sites were constructed into pmirGLO vectors (Promega Corporation, Fitchburg, WI, USA), and luciferase reporters were analyzed. Vectors, NC mimics, and miR-492 mimics were cotransfected into cells using Lipofectamine 2000 (Invitrogen, Thermo Fisher Scientific). After 48 h transfection, the luciferase activity was detected by dual-luciferase reporter assay (Promega Corporation).

### 2.9. Statistical Analysis

Data were analyzed using SPSS 15.0 software (IBM, USA) and presented as mean ± standard deviation. The difference between the two groups was determined using Student's *t*-test. A *p* value of <0.05 was identified as statistically significant.

## 3. Results

### 3.1. Sequence and Structure of circFBXW7

**Figure 1:**
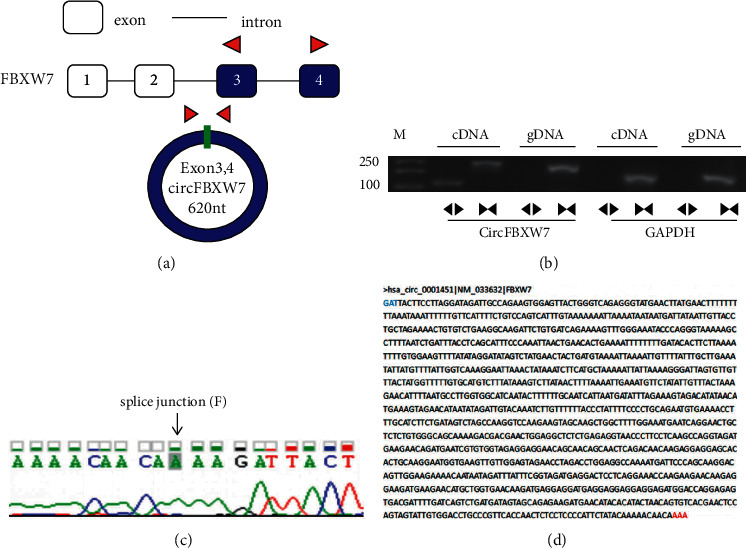
The sequence and structure of circFBXW7. (a) circFBXW7 contains 620 nucleotides and was derived from exons 3 and 4 of *FBXW7*. (b) Divergent and convergent primers designed for PCR to validate the structure of circFBXW7. *GAPDH* as control. (c) The splice junction of circFBXW7. The products of divergent primers were collected for Sanger sequencing. (d) The sequence of circFBXW7 in circBase (https://circrna.org/). FBXW7, F-box and WD repeat domain containing 7; M, DNA marker; cDNA, complementary DNA; GAPDH, glyceraldehyde-3phosphate dehydrogenase.

We first confirmed the origin and the structure of circFBXW7, which consisted of 620 nucleotides and was derived from exons 3 and 4 of *FBXW7* (Figure 1(a)). Divergent and convergent primers were designed to validate the structure of circFBXW7; divergent primers amplify products from cDNA but not from genomic DNA (Figure 1(b)). Sanger sequencing of the amplified products verified the splice junction and the circular structure of circFBXW7 (Figure 1(c)). The sequence in circBase (https://circrna.org/) is shown in Figure 1(d). The above results indicated that circFBXW7 was a closed loop transcribed from exons 3 and 4 of *FBXW7*.

### 3.2. Overexpression of circFBXW7 Reduced Cell Proliferation, Migration, and Invasion

**Figure 2:**
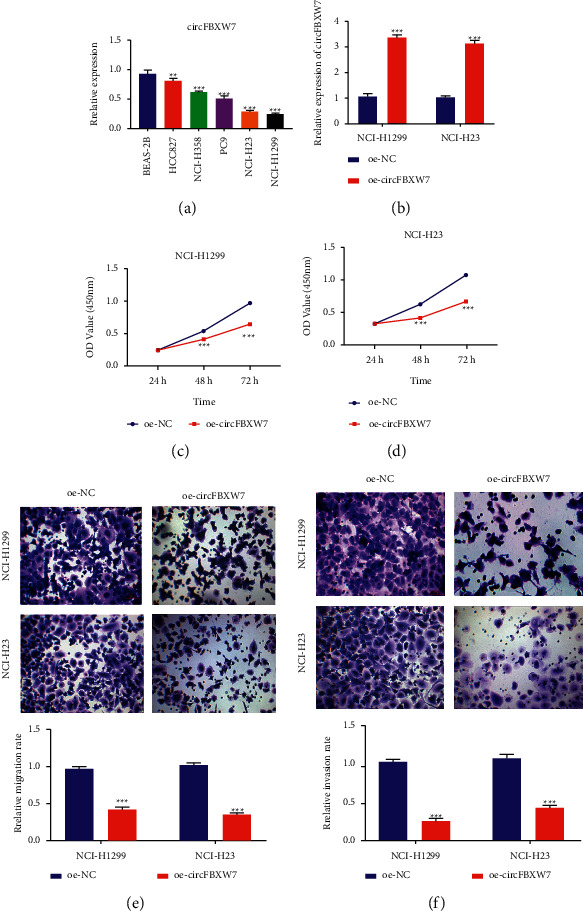
Overexpression of circFBXW7 reduced cell proliferation, migration, and invasion. (a) circFBXW7 downregulated in NSCLC cells, including HCC827, PC9, NCI-H23, and NCI-H1299 cells. qRT-PCR was used to test the expression of circFBXW7. (b) circFBXW7 expression upregulated by oe-circFBXW7 transfection for 48 h in NCI-H23 and NCI-H1299 cells. ((c) and (d)) Overexpression of circFBXW7 decreased proliferation in NCI-H1299 and NCI-H23 cells; CCK-8 was used to assess cell proliferation. ((e) and (f)) Overexpression of circFBXW7 reduced the migration and invasion ability of NCI-H1299 and NCI-H23 cells; Transwell assays were used to assess cell migration and invasion (40x). FBXW7, F-box and WD repeat domain containing 7; oe-NC, circFBXW7 overexpression negative control; oe-circFBXW7, circFBXW7 overexpression plasmids; qRT-PCR, real-time quantitative polymerase chain reaction; CCK-8, cell counting kit-8; ^*∗∗∗*^*p* < 0.001.

To examine the expression of circFBXW7 in NSCLC, we conducted qRT-PCR. As shown in Figure 2(a), expression of circFBXW7 was downregulated in NSCLC cells (HCC827, PC9, NCI-H23, and NCI-H1299) compared with that in normal human lung epithelial BEAS-2B cells. NCI-H23 and NCI-H1299 cells were determined to have the lowest expression of circFBXW7 and were selected for functional study. The overexpression plasmids were transfected into these cells for 48 h, followed by qRT-PCR to assess the efficiency of overexpression of circFBXW7. The results showed that the expression of circFBXW7 was significantly upregulated in the overexpression group, which was more than 3 times that of the control group. Following overexpression-circFBXW7 (oe-circFBXW7) transfection, the level of circFBXW7 was significantly increased compared with that in the oe-NC-treated group (Figure 2(b)). CCK-8 results showed that overexpression of circFBXW7 reduced cell proliferation (Figures 2(c) and 2(d)) and colony formation (Supplement Figure 1). In addition, overexpression of circFBXW7 reduced migration and invasion of NCI-H23 and NCI-H1299 cells in Transwell assays (Figures 2(e) and 2(f)). The above results indicated that circFBXW7 expression was downregulated in NSCLC and that overexpression of circFBXW7 reduced cell proliferation, migration, and invasion of NSCLC cells.

### 3.3. Bioinformatics Analysis and Preliminary Verification of circFBXW7 Downstream Target miRNA

**Figure 3:**
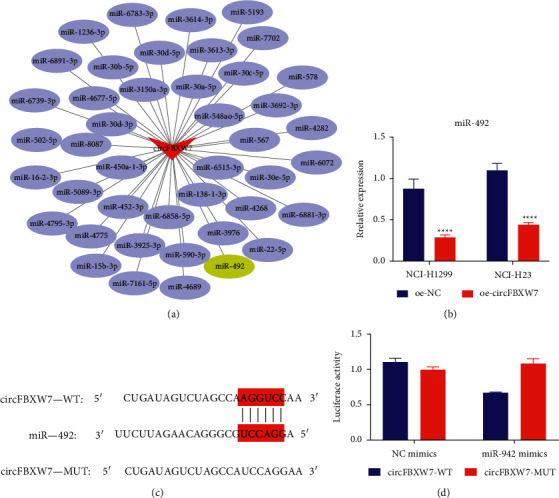
CircFBXW7 targeted miR-492. (a) A regulatory network of circFBXW7-miRNAs constructed by Cytoscape. Potential miRNAs of circFBXW7 were predicted by miRanda software and miTarBase database. (b) The expression of miR-492 downregulated after circFBXW7 overexpression in both NCI-H23 and NCI-H1299 cells; qRT-PCR validated miR-492 expression. (c) The binding sites of circFBXW7 and miR-492. (d) The targeted binding relationship between circFBXW7 and miR-492 verified by a dual-luciferase reporter assay. FBXW7, F-box and WD repeat domain containing 7; oe-NC, circFBXW7 overexpression negative control; oe-circFBXW7, circFBXW7 overexpression plasmids; qRT-PCR, real-time quantitative polymerase chain reaction; ^*∗∗∗*^*p* < 0.001.

To explore the potential mechanism of action of circFBXW7 in NSCLC, we predicted the potential miRNAs of circFBXW7 by miRanda software and miTarBase database and plotted circRNA-miRNA interaction using Cytoscape software (Figure 3(a)). Among the miRNAs, miR-492 has been reported to regulate NSCLC progression [18–20]. Then, qRT-PCR detection showed that miR-492 expression was significantly downregulated in the oe-circFBXW7 group (Figure 3(b)).  Figure 3(c) shows the binding site of circFBXW7 and miR-492. Dual-luciferase detection revealed that the fluorescence activity of the circFBXW7 WT plasmid group was significantly downregulated, confirming the targeted binding sites between circFBXW7 and miR-492 (Figure 3(d)).

### 3.4. CircFBXW7 Reduced Cell Proliferation, Migration, and Invasion through Binding to miR-492

**Figure 4:**
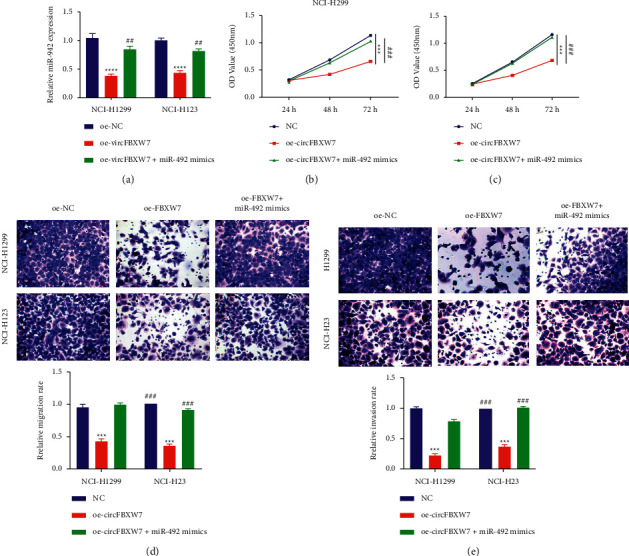
CircFBXW7 reduced cell proliferation, migration, and invasion by regulating miR-492. (a) The expression of miR-492 was downregulated by circFBXW7 overexpression, whereas this effect was reversed by miR-492 mimics in NCI-H23 and NCI-H1299 cells; the expression of miR-492 was determined by qRT-PCR after transfection for 48 h. ((b) and (c)) Proliferation was reduced by circFBXW7 overexpression, whereas this effect was reversed by miR-492 mimics in NCI-H23 and NCI-H1299 cells; the proliferation of NCI-H23 and NCI-H1299 cells was detected at 24, 48, and 72 h by CCK-8 assay after transfection for 48 h. ((d) and (e)) migration and invasion were decreased by circFBXW7 overexpression, whereas this effect was reversed by miR-492 mimics in NCI-H23 and NCI-H1299 cells. Migration and invasion were detected via Transwell assay after transfection for 48 h (40x). FBXW7, F-box and WD repeat domain containing 7; oe-NC, circFBXW7 overexpression negative control; oe-circFBXW7, circFBXW7 overexpression plasmids; qRT-PCR: real-time quantitative polymerase chain reaction; CCK-8: cell counting kit-8; ^*∗∗∗*^*p* < 0.001 vs. the NC group, ^*∗∗∗*^*p* < 0.001 vs. the oe-circFBXW7 group.

To confirm whether the inhibitory effect of circFBXW7 overexpression on proliferation, migration, and invasion was regulated by miR-492, we synthesized miR-492 mimics and verified its effect via qRT-PCR. As shown in Figure 4(a), the expression of miR-492 was repressed when circFBXW7 was overexpressed, whereas this was reversed by miR-492 mimics in NCI-H23 and NCI-H1299 cells, indicating that the synthesized mimics effectively promoted the expression of miR-492. The proliferation of NCI-H23 and NCI-H1299 cells was promoted in the oe-circFBXW7-treated group in the presence of the miR-492 mimics compared with that in the oe-circFBXW7-treated group (Figures 4(b) and 4(c)). In addition, transfection of miR-492 mimics rescued the inhibition of circFBXW7 overexpression on cell migration and invasion (Figures 4(d) and 4(e)). These results showed that circFBXW7 could reduce cell proliferation, migration, and invasion by regulating miR-492.

## 4. Discussion

NSCLC is a common type of lung cancer characterized by poor prognosis [21]. CircRNA plays an important role in the occurrence and development of NSCLC, but its pathogenesis in NSCLC has not been fully clarified. In this study, we showed that circFBXW7 was expressed at a low level in NSCLC cells, particularly in NCI-H23 and NCI-H1299 cells. Overexpression of circFBXW7 in NCI-H23 and NCI-H1299 cells suppressed cell proliferation, migration, and invasion. However, following the overexpression of circFBXW7, the expression of miR-942 was downregulated in NCI-H23 and NCI-H1299 cells. MiR-942 mimics reversed the effect of circFBXW7 overexpression on cell proliferation, migration, and invasion in oe-circFBXW7-treated NCI-H23 and NCI-H1299 cells.

*FBXW7* is the parent gene of circFBXW7, which has been reported to be involved in cell division, growth, migration, invasion, and drug resistance [22–24]. CircFBXW7 is transcribed from exons 3 and 4 of *FBXW7* and has been reported to be vital in various biological processes. For instance, the expression of circFBXW7 was reported to be downregulated in tumor tissues, and overexpression of circFBXW7 significantly inhibited the proliferation, migration, and invasion ability of triple-negative breast cancer and glioma [12, 25]. It was reported that circFBXW7 attenuated the malignant progression of lung adenocarcinoma by sponging miR-942-5p [26]. Here, we observed that circFBXW7 was expressed at a low level in NSCLC cells. Overexpression of circFBXW7 decreased NSCLC cell proliferation, migration, and invasion. This is the first report on the role of circFBXW7 in NSCLC. This finding suggests that circFBXW7 could be a marker for the diagnosis of NSCLC.

CircRNAs have been reported to act as sponges for miRNAs to regulate mRNA expression and translation [27]. Therefore, we screened potential target miRNAs as predicted by miRanda software and miTarBase database combined with the results from previous studies. MiR-942 was selected for further study [18–20]. Zhang et al. reported that overexpression of hsa_circ_0005567 promoted polarization of M2-type macrophages through the miR-492/SOCS2 axis and inhibited the progression of osteoarthritis [28]. In addition, circ_0001368 inhibited the growth and invasion of renal cell carcinoma by inhibiting miR-492 [29]. Hsa_circ_0072309 inhibited the proliferation and invasion of breast cancer cells by targeting miR-492 [30]. Ye et al. suggested that overexpression of circFBXW7 inhibited triple-negative breast cancer progression via sponging of miR-197-3p [25]. In our study, we observed that circFBXW7 targeted miR-492. We verified that overexpression of circFBXW7 decreased the expression of miR-492 in both NCI-H23 and NCI-H1299 cells. In addition, the inhibitory effect of circFBXW7 overexpression on cell proliferation, migration, and invasion was reversed by miR-492 mimics. This finding suggests that circFBXW7 plays an important role in NSCLC by regulating miR-492.

However, there are some limitations in this study. More works are needed to verify the role of circFBXW7 in NSCLC, such as examining the expression of circFBXW7, miR-942 in NSCLC tissues, and validating the relationship between circFBXW7 and miR-942 in vivo.

Taken together, this study suggests that overexpression of circFBXW7 inhibits miR-942 expression, which, in turn, inhibits the progression of NSCLC. CircFBXW7 could be a promising biomarker for lung cancer patients for predicting tumor prognosis and recurrence.

## Figures and Tables

**Table 1:** The primer sequences in this study.

Name	Sequences (5′-3′)
circFBXW7-CF	CCAGTAGTATTGTGGACCTGCCC
circFBXW7-CR	CCCTCTGACCCAGTAACTCCACT
circFBXW7-LF	TGTGTCTGAAGGCAAGATTCTGT
circFBXW7-LR	CAGTTAATTTGGGAAATGCTGAGGT
GAPDH-F	GAGTCAACGGATTTGGTCGT
GAPDH-R	GACAAGCTTCCCGTTCTCAG
miR-492-F	AACAATAGGACCTGCGGGAC
miR-492-RT	GTCGTATCCAGTGCAGGGTCCGAGGTATTCGCACTGGATACGACAAGAAT
Universe-R	GTGCAGGGTCCGAGGT
U6-F	CTCGCTTCGGCAGCACA
U6-R	AACGCTTCACGAATTTGCGT

## Data Availability

The sequence of hsa_circ_0001451 comes from circBase, and the products of divergent primers were used for Sanger sequencing to validate the circularity of circRNA. The datasets generated and/or analyzed during the current study are available in the circBase repository.

## Supplementary Materials

Supplementary Materials Supplement Figure 1. circFBXW7 reduced cell colony formation. FBXW7, F-box and WD repeat domain containing 7; oe-NC, circFBXW7 overexpression negative control; oe-circFBXW7, circFBXW7 overexpression plasmids; ^*∗∗*^*p* < 0.01.
