# Supplementary figures and images for: CircFBXW7 Inhibits Proliferation, Migration, and Invasion of Nonsmall Cell Lung Cancer Cells by Regulating miR-492
Source: J Oncol. 2022 Sep 8;2022:8699359. doi: 10.1155/2022/8699359 (PMC9477579; doi:10.1155/2022/8699359)

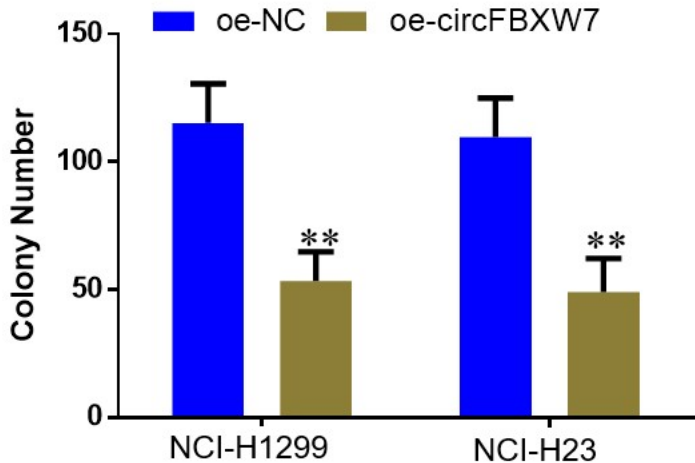

Supplement: Supplementary Materials — Supplement Figure 1. circFBXW7 reduced cell colony formation. FBXW7, F-box and WD repeat domain containing 7; oe-NC, circFBXW7 overexpression negative control; oe-circFBXW7, circFBXW7 overexpression plasmids; ∗∗p < 0.01. [file 8699359.f1.pdf]
